# AI-driven early detection of severe influenza in Jiangsu, China: a deep learning model validated through the design of multi-center clinical trials and prospective real-world deployment

**DOI:** 10.3389/fpubh.2025.1610244

**Published:** 2025-08-18

**Authors:** Yifei Chen, Yan Bo

**Affiliations:** ^1^The Department of Emergency Medicine, The Affiliated Hospital of Yangzhou University, Yangzhou University, Yangzhou, China; ^2^The Department of Medicine, Northwest Minzu University, Lanzhou, China

**Keywords:** severe influenza, deep learning, clinical decision support, predictive modeling, multi-center trial, artificial intelligence, research design

## Abstract

**Background:**

Influenza-related global deaths reach 650,000 annually. The current highly lethal clinical subtype of influenza is severe influenza.

**Aim:**

To develop and validate a deep learning based model for early diagnosis of severe influenza.

**Methods:**

This is a multi-centre, double-blind, multi-stage, randomised controlled clinical trial. We initially developed a framework for a 5-phase study: model development, external validation, multi-reader study, randomised controlled trial and prospective validation. The data source for the preview programme is electronic health record data from 87 hospitals in Jiangsu Province from 2019 to 2025.

**Significance:**

Our expected result is that the developed model of severe influenza can be more accurate and have a lower misdiagnosis rate than traditional clinical assessment. The pre-specified AUC was 0.18 (95% CI: 0.14-0.22), with an expected 32% reduction in misdiagnosis. The model’s performance was consistent across patients in older adults, underlying disease, and resource-poor areas. The added value of the study is that it is effective in improving early recognition of severe influenza.

**Ethics and dissemination:**

This study was approved by the Institutional Review Board of Yangzhou University Hospital (IRB No. YKL08-002). Written informed consent was obtained from all participants. The results of this study will be disseminated in the form of a conference in the Jiangsu Province area, which will facilitate the translation of clinical research results and provide a powerful decision-making tool for the precise prevention and control of severe influenza.

**Clinical trial number:**

https://clinicaltrials.gov/, identifier (ChiCTR2000028883).

## 1 Introduction

Influenza causes 650,000 deaths annually and remains a threat to global health. Influenza has a mortality rate of approximately 20 per cent, and deaths due to influenza predominantly occur among infants, pregnant women, the elderly and the immunocompromised (1, 2). Although progress has been made with previously developed predictive tools and antiviral therapies, this has not reduced mortality from severe influenza (3). A key issue is the dynamic gap between clinical decision-making and risk of severe influenza. Evidence suggests that physicians are overconfident in the outcome of subjective clinical assessments leading to misdiagnosis and quality delays in severe influenza. Another factor, of course, arises from differences in the definition of ‘cure’ in the healthcare system (4, 5). A study by Valenzuela-Sánchez et al. (2024) reported that 34% of severe influenza cases were initially incorrectly classified as mild. The reason for this was an over-reliance on non-specific symptoms by clinicians when assessing influenza (6). This result implies that we need more objective and accurate tools to improve the accuracy of clinical judgement.

Artificial Intelligence (AI) can learn deeply from human health data so that closed-source predictive models can be constructed to identify high-risk patients. The current intersection of AI and healthcare data has shown revolutionary potential (7, 8). Yang et al. (2023) developed an influenza prognostic AI model using only city-scale data, which has achieved a performance AUC value of 0.89 (9). Of course, the lack of external validation of models constructed using AI across regions and healthcare resources means that more effort is needed for AI to address real-world clinical work (10). Dai et al. (2024) concluded that constructed AI diagnostic tools do not work well when put into prospective clinical practice due to population heterogeneity and data drift (11). This may be due to the lack of research-oriented personnel in the field of AI, leaving little understanding of dynamic risk prediction in high-risk subgroups in a clinical context (12).

The aim of our research is to develop and validate a deep learning-based diagnostic model for severe influenza. In order to be able to specify the research tasks at each stage, we established a methodological framework.

1. Data: data were obtained from 87 hospitals in Jiangsu Province, China. The data features of interest were clinical information, laboratory test results, and demographic information.
2. Stages: 5 research phases with model building, internal validation, comparative study, randomised trial, and prospective real-world validation.
3. Collaboration: the aim of the model is to assist clinicians in accurately identifying patients with severe influenza.

We hypothesize that this model will significantly improve AUC (*Δ* > 0.15) compared to physician-only assessments, reduce misdiagnosis rates by ≥30%, and maintain robustness across hospitals with varying resource levels. By addressing both algorithmic and human factors, this research seeks to establish a scalable solution for mitigating influenza-related mortality, particularly in resource-constrained settings.

## 2 Methods

### 2.1 Study setting

Yifei Chen of the Affiliated Hospital of Yangzhou University proposed the research concept of stratified prevention of severe influenza in 2019, and this was subsequently supported by Yan Bo of Northwest Minzu University in October 2022.

This is a multi-center, multi-phase clinical trial. The study will be conducted across 87 tertiary general hospitals in all prefecture-level cities of Jiangsu Province, China. This study is a continuation of the 2019 investigation of influenza A (ChiCTR2000028883) and is registered on the Open Science Framework (OSF) registry platform (13). This clinical trial will be reported according to Standard Protocol Items Recommendations for Interventional Trials (SPIRIT) 2013 standard specifications (14).

#### 2.1.1 Hospital inclusion criteria

We have pre-set criteria for hospital eligibility for the study. Hospitals must be accredited tertiary general hospitals, which provide full emergency and inpatient services. Hospitals should have at least 100 patients with influenza diagnosed by laboratory testing between 2021 and 2023. Hospital clinicians must use electronic health records (EHRs) so that symptoms of influenza patients and other information used for clinical research can be recorded in a standardised format. Longitudinal, anonymous data sharing programmes must be in place within hospitals to ensure the security of patient and clinician information.

#### 2.1.2 Rationale for multi-center design

The principle of the multicentre design is based on a statistically full sample size as a reference basis. In other words, we included all hospitals in Jiangsu Province that met the study eligibility criteria. This design allows for the establishment of model performance in different Jiangsu regions and in different clinical settings, and ensures general applicability from the statistical principle. Considering the perspective of urban and rural stratification, healthcare resources are generally richer in cities than in rural areas, and we designed it in this way to precisely address healthcare inequality under the geographic factor. Dr Yifei Chen concluded that influenza outbreaks have a seasonal element, and the peaks of outbreaks in the northern part of Jiangsu are different from those in the southern part of Jiangsu. This design in a full sample size perspective provides a natural experimental platform for assessing influenza disease observations with seasonal fluctuations. We ensure that the accuracy of the diagnosis will not be affected by the transmission factors of resource differences and geographical differences.

#### 2.1.3 Trial registration and reporting standards

**Figure 1:**
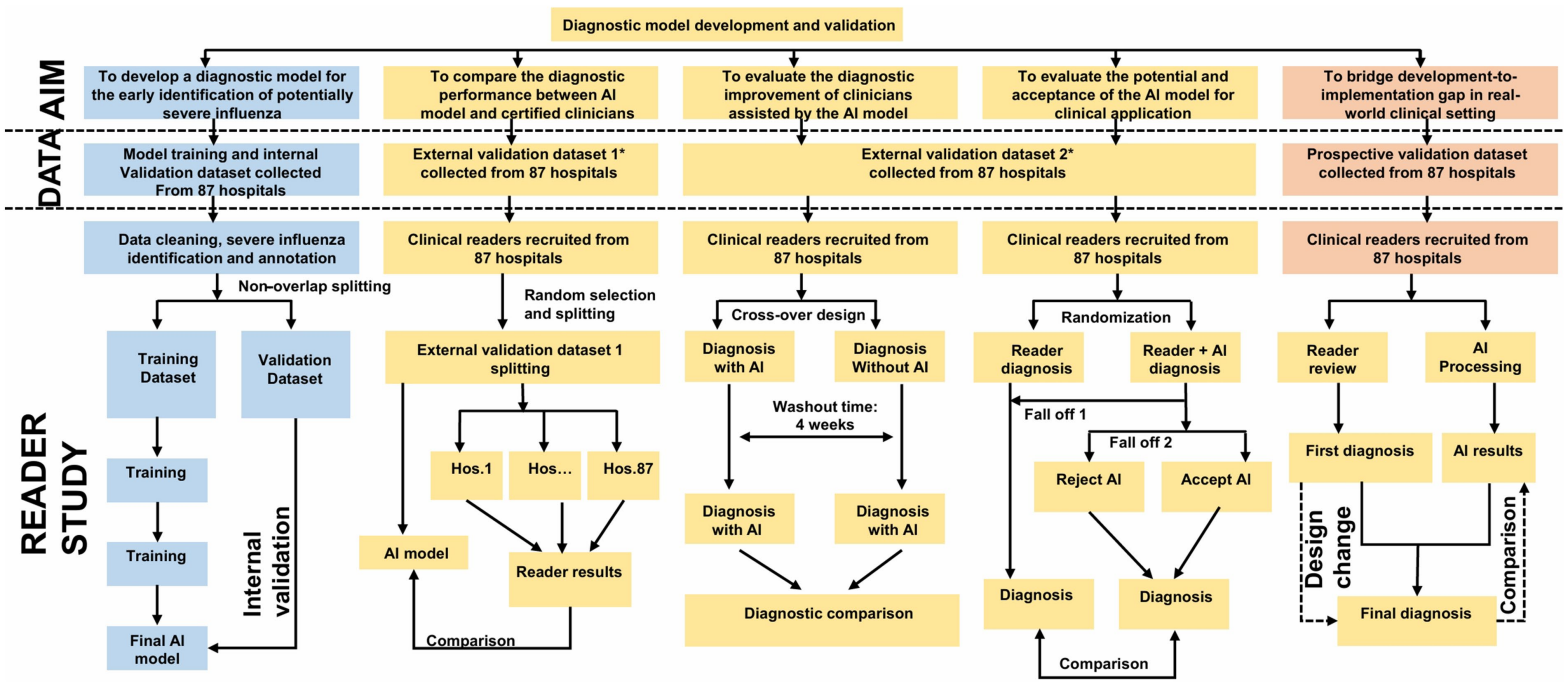
Study overview. Flowchart illustrating the development and validation of a diagnostic model for early identification of severe influenza. The process involves collecting datasets from 87 hospitals, dividing them for training and validation, and comparing AI and clinician diagnoses. It includes internal and external validation steps, randomization, reader studies, and comparisons between AI-assisted diagnoses and clinician diagnoses, with design change considerations in the prospective validation phase. This figure is drawn by by Figdraw.

This study was registered with the OSF platform (DOI: 10.17605/OSF.IO/SC93Y) and the China Clinical Trial Registry (ChiCTR2000028883). The study protocol was developed following the SPIRIT 2013 guidelines (15). The study phase design we used a visual presentation (Figure 1). This dual registration and standardised reporting framework enhanced the reproducibility of the study.

#### 2.1.4 Phase overview

The study comprises five sequential phases, which is based on the multiple nesting approach used in Yan Bo’s past methods (16–20).

1. Model development: retrospective data (2019–2024) from 87 hospitals for training and internal validation.
2. External validation-comparative study: benchmarking model performance against clinician diagnoses.
3. Multi-reader, multi-case validation: assessing inter-physician variability with/without model assistance.
4. Randomized controlled trial: evaluating real-time diagnostic accuracy in a clinician-AI collaborative setting.
5. Prospective validation: real-world testing during the 2025 influenza season.

### 2.2 Eligibility criteria

#### 2.2.1 Inclusion criteria for patients

1. Laboratory-confirmed influenza virus infection via nucleic acid testing [reverse transcription polymerase chain reaction (RT-PCR)] or rapid antigen testing, adhering to China’s *Diagnosis and Treatment Protocol for Influenza*;
2. All age groups;
3. Written consent obtained from patients: (a) patients with full civil capacity (more than 18 years); (b) Legal guardians for minors (less than 18 years), incapacitated adults, or patients with cognitive impairment.

#### 2.2.2 Exclusion criteria for patients

1. Co-infection with other respiratory pathogens confirmed by multiplex polymerase chain reaction (PCR);
2. Critical illness unrelated to influenza at enrollment;
3. Incomplete baseline data (such as missing symptom onset date, laboratory results).

#### 2.2.3 Inclusion criteria for clinicians

1. less than 5 years of clinical experience in influenza diagnosis or participation in national-level influenza research projects;
2. Willingness to complete protocol-mandated tasks, including dual-round diagnosis (baseline vs. model-assisted) and post-study interviews;
3. Currently practicing in a tertiary general hospital in Jiangsu Province.

#### 2.2.4 Exclusion criteria for clinicians

1. Temporary or part-time staff without direct patient care responsibilities;
2. Participation in conflicting AI-related clinical trials within the past 6 months.

### 2.3 Interventions

#### 2.3.1 Phase I: model development and internal validation

This phase utilizes retrospective EHRs from 87 hospitals (Table 1) in Jiangsu Province (from January 2019 to December 2024), encompassing four key data categories:

1. Demographics: age, sex, vaccination history, and comorbidities.
2. Clinical symptoms: fever duration, cough frequency, dyspnea severity, and other symptoms detailed (Table 2).
3. Laboratory/imaging data: viral subtype (confirmed via RT-PCR), blood cell counts, and chest X-ray/computed tomography (CT) findings.
4. Outcomes: severe influenza progression and mortality, defined as intensive care unit (ICU) admission or mechanical ventilation.

**Table 1:** Datasets distribution.

City	Hospitals
Nanjing	Jiangsu Provincial People’s Hospital, General Hospital of the Eastern Theater Command, Nanjing Drum Tower Hospital, Jiangsu Provincial Hospital of Traditional Chinese Medicine, Zhongda Hospital Affiliated to Southeast University, Nanjing First Hospital, The Second Affiliated Hospital of Nanjing Medical University, Nanjing Hospital of Traditional Chinese Medicine
Wuxi	Wuxi People’s Hospital, The Affiliated Hospital of Jiangnan University, Wuxi Hospital of Traditional Chinese Medicine
Xuzhou	The Affiliated Hospital of Xuzhou Medical University, Xuzhou Central Hospital, Xuzhou First People’s Hospital, Xuzhou Mining Group General Hospital, Xuzhou Hospital of Traditional Chinese Medicine
Changzhou	Changzhou First People’s Hospital, Changzhou Hospital of Traditional Chinese Medicine, The Third Affiliated Hospital of Soochow University (Changzhou Second People’s Hospital)
Suzhou	The First Affiliated Hospital of Soochow University, The Second Affiliated Hospital of Soochow University, Suzhou Municipal Hospital, Suzhou Hospital of Traditional Chinese Medicine
Nantong	The Affiliated Hospital of Nantong University, Nantong First People’s Hospital, Nantong Hospital of Traditional Chinese Medicine
Lianyungang	Lianyungang First People’s Hospital, Lianyungang Hospital of Traditional Chinese Medicine, Lianyungang Second People’s Hospital
Huai’an	Huai’an First People’s Hospital, Huai’an Second People’s Hospital, Huai’an Hospital of Traditional Chinese Medicine
Yancheng	Yancheng First People’s Hospital, Yancheng Hospital of Traditional Chinese Medicine, Yancheng Third People’s Hospital
Yangzhou	Subei People’s Hospital, Yangzhou First People’s Hospital, Yangzhou Hospital of Traditional Chinese Medicine
Zhenjiang	The Affiliated Hospital of Jiangsu University, Zhenjiang First People’s Hospital, Zhenjiang Hospital of Traditional Chinese Medicine
Taizhou	Taizhou People’s Hospital, Taizhou Hospital of Traditional Chinese Medicine, Taixing People’s Hospital
Suqian	Suqian People’s Hospital, Suqian Hospital of Traditional Chinese Medicine

**Table 2:** Data collection framework.

Category	Specific data items	Collection tool
Demographics	Age, sex, contact details, vaccination history, comorbidities	EHR extraction
Symptoms	Fever (duration, max temperature), cough (frequency, sputum), dyspnea, headache, nausea/vomiting	Structured EHR forms + Patient diaries
Medical History	Previous influenza episodes, chronic diseases (type, duration), vaccination details	EHR extraction + Clinician interview
Laboratory/Imaging	Viral subtype (RT-PCR), blood counts, chest X-ray/CT findings	Lab/radiology reports
Treatment	Antiviral drugs (name, dosage), hospitalization duration, ICU admission	EHR extraction + Pharmacy records
Outcomes	Severe influenza progression (yes/no), recovery time, mortality	Adjudication panel review

We plan to divide the collected data into an 80% training subset and a 20% internal validation subset. When building the model, we will progressively stratify by hospital, influenza season, and high-risk subgroup characteristics, so that the initial model built may have a balanced representation. We plan to build a preliminary severe influenza model using the deep neural network model of ResNet-50 (21). The metrics we predesigned to assess the baseline performance of the model were area under the curve (AUC) and sensitivity. Whereas the complex technical parameters of these will be published in the final version of the research results, in this research protocol we focused more on the deployment of the clinical research programme. This is more beneficial for clinicians or influenza patients to read and disseminate.

#### 2.3.2 Phase II: external validation-comparative study

The goal of the second phase of the task was to comparatively confirm the diagnostic accuracy of the additional diagnostic assessment using the AI models built in the first phase to assist clinicians in diagnostic assessment. We categorised clinicians into residents, attending physicians and chief physicians according to their experience level. We asked clinicians in the control group to independently judge these influenza cases without the assistance of the AI model; while clinicians in the experimental group did the diagnostic assessment of these influenza cases with the assistance of the AI model. Under this comparison, the metrics of AUC, sensitivity, specificity, and diagnostic consumption time are what are used to initially evaluate the performance of the AI model. Imagine the exciting news that an AI model-assisted clinician can complete a diagnostic assessment with 95% accuracy in just 10 minutes. This dual assessment framework quantifies the added value of AI models in the real world.

#### 2.3.3 Phase III: multi-reader, multi-case validation

Phase 3 used a two-round diagnostic design for evaluating the impact of AI model assistance on the effectiveness of clinician decision-making. The AI models used in Phase 3 were calibrated in the Phase 2 validation phase. Recruited clinicians are split into two rounds subsequently evaluating cases independently then filling in the diagnosis.

The first round of tasks that clinicians receive is the baseline diagnostic assessment. In the baseline diagnostic assessment, clinicians are required to evaluate cases using a standard influenza diagnostic protocol and subsequently complete the diagnostic results.

The second round of tasks allows clinicians to access the risk scores and key influencing factor scores generated by the AI model to fill in the diagnosis during the evaluation of the case.

All clinicians were required to undergo two rounds of tasks. This was done with reference to a previous study (22). The use of machine-assisted diagnosis by clinicians can increase their productivity (23). To minimise bias, cases are randomly reordered between rounds of assessment and a 4-week washout period is set between assessments to reduce recall effects. Diagnostic accuracy (measured as AUC) and clinician confidence (scored on a 5-point Likert scale) will be compared between rounds of assessment to quantify the impact of the model on decision consistency and clinician certainty. This multi-reader, multi-case framework ensures robust assessment of AI-human collaboration while addressing variations in real-world clinical work.

#### 2.3.4 Phase IV: randomized controlled trial

This phase is a pragmatic randomised controlled design to assess the effectiveness of AI-assisted diagnosis in the real world. Clinicians will be assigned in a 1:1 ratio to either the control group (using standard clinical assessment protocols) or the AI model-assisted group. Allocation will be by block group randomisation, stratified by hospital level (urban vs. rural) and clinician experience level (resident, attending, chief) to ensure balanced representation in key subgroups.

The control group will rely exclusively on routine diagnostic workflows, while the model-assisted group will integrate AI outputs into their decision-making process and retain full authority to accept or reject recommendations. The primary outcome is the between-group AUC difference, with a ΔAUC greater than 0.15 defined as clinically significant, which is consistent with the U.S. Food and Drug Administration’s (FDA) benchmarks for diagnostic tools based on artificial intelligence or machine learning (AI/ML). The design simulates real-world clinical practice and quantifies the added value of AI in improving diagnostic accuracy while preserving clinician autonomy.

#### 2.3.5 Phase V: prospective validation study

As Dai et al. illustrate the point, using prospective cohort data validation provides a better view of the model’s performance in the real world (24). This final phase evaluates the model’s real-world clinical utility during the 2025 influenza season. The AI model will be deployed in real time to analyze EHRs of newly admitted influenza patients across all 87 hospitals. Clinicians will receive model-generated predictions (such as risk scores, key contributing factors) within 2 h of case entry via a secure, institution-specific dashboard.

Data collection will capture:

1. Clinician actions: Initial diagnosis (pre-model assessment), final diagnosis (post-model review), and time-to-decision (minutes from case entry to diagnosis).
2. Model performance: Concordance between model predictions and ground truth (severe influenza confirmed by an independent adjudication panel).

This prospective design validates the model’s operational feasibility, diagnostic timeliness, and accuracy in dynamic clinical environments, providing critical evidence for scalability and regulatory approval.

### 2.4 Outcomes

#### 2.4.1 Primary outcome

As the ultimate goal of this clinical trial is to create highly accurate AI models. Therefore the primary outcome metric was set as the area under the curve (AUC) (25). The AUC quantifies the discriminatory ability of the AI model so that it can distinguish patients who may develop severe influenza from those who will not. Since calculating the AUC requires the use of sensitivity, specificity, true positive rate, true negative rate, false positive rate, and false negative rate, more details describing these results can be found in our separate studies (16, 20).

To account for differences in diagnostic assessments after multiple clinicians read the cases, we planned to use the Dorfman-Berbaum-Metz (DBM) method. The DBM adjusts for differences in diagnostic assessments by clinicians so that AUC difference values between the two groups can be estimated (22). ROC curves were calculated using the pROC (version 1.18.0) software package (28). The AUC values as well as their 95% confidence intervals will then be calculated using MRMCaov (version 0.2.1) (27). For differences between the experimental and control groups, we expected to use the Mann-Whitney U test (29). Clinically significant improvement was defined as an AUC difference between the experimental and control groups greater than 0.15. The null hypothesis here is that the AUC difference is less than 0.7, and the AI model has no discriminatory power. Our method of using AUC to set thresholds not only follows statistical patterns, but is also highly compliant with the criteria for the development of AI-driven diagnostic tools.

#### 2.4.2 Secondary outcomes

Secondary endpoints include diagnostic sensitivity and specificity, defined as the proportion of true severe influenza cases correctly identified by the model or clinicians, which reflects the tool’s capacity to minimize missed diagnoses in high-risk populations (30).

#### 2.4.3 Subgroup analyses

Predefined subgroup analyses will assess heterogeneity in model performance across key variables to evaluate the impact of local healthcare infrastructure and influenza epidemiology.

1. patient characteristics: age (infants, children, adults, older adults), comorbidities (cardiovascular disease, diabetes), and vaccination status (yes, no);
2. clinician factors: experience level (resident, attending, chief physician);
3. hospital resource tier: urban or rural facilities;
4. geographic variation: northern or southern regions of Jiangsu Province.

These analyses aim to identify subgroups where the model excels or underperforms, informing targeted clinical deployment. Statistical adjustments, such as Bonferroni correction for multiple comparisons and multivariable regression for confounders, will ensure robust interpretation of subgroup differences.

#### 2.4.4 Handling missing data

To address missing data, variables with more than 20% missing values will be excluded to ensure reliability. For remaining gaps, the Multivariate Imputation by Chained Equations (MICE) package in R will be employed, leveraging iterative modeling across variables to impute missing values under the missing-at-random assumption. This method preserves dataset integrity by estimating missing entries based on observed patterns, thereby minimizing bias while maintaining statistical power.

#### 2.4.5 Adjudication of ground truth

To ensure accurate outcome classification, an independent adjudication panel comprising three senior infectious disease specialists will retrospectively review all cases classified as severe influenza, verifying endpoints such as ICU admission, mechanical ventilation, or mortality. Discrepancies in outcome assessments among panel members will be resolved through majority vote, with detailed documentation of dissenting opinions. This rigorous process minimizes misclassification bias and enhances the reliability of outcome data, serving as the gold standard for model and clinician performance evaluation.

### 2.5 Sample size

#### 2.5.1 Calculation rationale

The sample size was determined based on the primary outcome (AUC comparison between model-assisted and clinician-only diagnoses) using a two-group superiority design. Key parameters were derived from prior studies on AI diagnostic tools (31, 32):

Expected effect size: ΔAUC = 0.15 (clinically meaningful improvement).

Type I error (*α*): 0.05 (two-tailed).

Power (1-*β*): 0.95.

Attrition rate: 20% adjustment for patient dropout or incomplete data.

The minimum sample size for AUC comparison was calculated using the following formula (33):


$$n=\frac{{\left(Z_{1-\alpha /2}+Z_{1-\beta }\right)}^{2}\times [\mathrm{AU}C_{1}(1-\mathrm{AU}C_{1})+\mathrm{AU}C_{2}(1-\mathrm{AU}C_{2})]}{{\left(\mathrm{AU}C_{1}-\mathrm{AU}C_{2}\right)}^{2}}$$

Where:

$\mathrm{AU}C_{1}$ (model-assisted): 0.85 (34).

$\mathrm{AU}C_{2}$ (clinician-only): 0.70 (null hypothesis).

$Z_{1-\alpha /2}$ = 1.96, $Z_{1-\beta }$ = 1.645.

#### 2.5.2 Sample size derivation

The sample size calculation began with an initial estimate of 145 patients per group (290 total), derived from a power analysis targeting a clinically significant ΔAUC of 0.15 (*α* = 0.05, *β* = 0.95). To account for potential 20% attrition due to incomplete data or participant dropout, the final patient sample was adjusted to 174 per group (348 total). For clinicians, a minimum of 218 participants (109 per group) was required to ensure balanced representation across experience levels (resident, attending, chief) while addressing hospital-level clustering effects, as recommended by methodology for cluster-randomized trials (35). This dual-tiered approach balances statistical precision with pragmatic considerations, ensuring robust evaluation of the AI model’s impact in diverse clinical environments.

#### 2.5.3 Final allocation

The study will enroll a total of 348 patients, comprising both retrospective cohorts (historical data from 2019 to 2024) and prospective cohorts (real-time data from the 2025 influenza season). Clinician participation includes 218 practitioners, stratified by hospital tier (urban vs. rural) and seniority level (resident, attending, or chief physician) to ensure balanced representation of clinical expertise and institutional resource variability. This allocation strategy optimizes statistical power while reflecting real-world healthcare diversity, supporting robust validation of the AI model across heterogeneous settings.

#### 2.5.4 Justification

The patient sample size was determined through a power analysis to ensure ≥80% statistical power for detecting a clinically meaningful improvement (ΔAUC more than 0.15) in diagnostic accuracy, aligning with FDA guidelines for validating AI-driven medical devices (36).

### 2.6 Assignment of interventions

#### 2.6.1 Randomization and allocation

A computer-generated randomization sequence will be created using the blockrand package in R, stratified by hospital tier (urban vs. rural) and clinician experience level (resident, attending, or chief physician). This approach ensures balanced allocation across subgroups, minimizing confounding effects from institutional resources or practitioner expertise. Participants will be assigned to either the control group (standard clinical diagnosis) or the model-assisted group (AI-supported diagnosis) at a 1:1 allocation ratio. The randomization sequence will be concealed in sequentially numbered, opaque envelopes managed by an independent statistician to prevent selection bias, ensuring equitable distribution of participants and maintaining trial integrity.

To ensure unbiased group assignment, an independent statistician uninvolved in recruitment or data analysis will prepare opaque, sequentially numbered envelopes containing group allocations (control or model-assisted). These sealed envelopes will be securely stored and only opened sequentially by a dedicated study coordinator after clinicians complete baseline assessments. This procedure rigorously prevents selection bias by concealing allocation details until the point of intervention assignment, thereby safeguarding the randomization integrity and ensuring equitable distribution of participants across study groups.

#### 2.6.2 Blinding (masking)

To minimize bias, a single-blind design will be implemented for clinicians: those in the control group will remain unaware of their allocation status and the existence of the AI model, while clinicians in the model-assisted group will access predictions through a neutral interface labeled as a “Decision Support Tool,” avoiding explicit references to AI. Patients will be fully blinded to group assignments, with informed consent documents describing participation in “a study to improve influenza diagnosis” without disclosing AI involvement. Independent outcome adjudicators assessing severe influenza outcomes will also remain blinded to group allocations and model outputs, ensuring objective evaluation of clinical endpoints. This multi-layered blinding strategy safeguards against performance and detection bias, maintaining the trial’s scientific rigor.

#### 2.6.3 Intervention groups

In the control group, clinicians (without any AI assistance) will diagnose influenza cases using standard clinical protocols, integrating symptoms, laboratory results, and imaging findings. Patients in this group will receive routine care based solely on clinician assessments.

In contrast, the model-assisted group will utilize a secure digital dashboard providing real-time AI predictions, including a risk score (0–100%) for severe influenza progression and highlighted contributing factors (such as prolonged fever duration, abnormal lymphocyte counts).

While the model offers data-driven insights, its outputs are non-prescriptive; clinicians retain full authority to accept, modify, or reject recommendations, ensuring clinical judgment remains central to decision-making. This design balances technological support with practitioner autonomy, reflecting real-world diagnostic workflows.

### 2.7 Data collection

#### 2.7.1 Data sources and tools

The study spanned 5 years. A significant amount of data collection relied on EHRs and patient self-report diaries (Table 2). Two modes of sampling and supplementation were set up. Yifei Chen would randomly sample 5% of the data every month to check for completeness. Patients can supplement their data submissions through the WeChat app.

#### 2.7.2 Data collection timeline

The study’s data collection is structured into two distinct phases: retrospective (Phases I–II) and prospective (Phases III–V).

During the retrospective phase, EHRs data spanning 2019 to 2024 will be extracted from all 87 participating hospitals via institution-specific application programming interfaces (APIs), with completion targeted within 6 months to ensure timely progression to subsequent stages.

In the prospective phase, real-time data collection will occur throughout the 2025 influenza season, capturing clinical, diagnostic, and outcome variables as they emerge. These data will be synchronized daily to a centralized, secure database to maintain continuity and enable immediate analysis.

This phased approach balances historical insights with dynamic, real-world validation, ensuring comprehensive evaluation of the AI model across diverse temporal and operational contexts.

#### 2.7.3 Quality control measures

To ensure data accuracy and consistency, comprehensive quality control protocols were implemented. Standardized training was provided to clinicians and data entry staff, focusing on EHR documentation practices (such as symptom coding conventions and structured data entry). Automated validation rules were applied to detect anomalies, including range checks (such as body temperature less than 42°C) and logical consistency validations (such as ensuring symptom onset dates precede diagnosis dates). Additionally, manual audits were conducted by independent reviewers, who cross-validated 10% of randomly selected records against source documents (such as laboratory reports, imaging files) to verify data fidelity. These layered measures (combining education, technology, and human oversight) minimize errors and enhance the reliability of the dataset for robust model validation.

#### 2.7.4 Missing data handling

To minimize missing data, mandatory fields (such as viral subtype, symptom onset date) are enforced in digital entry forms, ensuring critical variables are consistently documented. For remaining missing values, multiple imputation via chained equations (MICE package) will be applied to continuous variables, leveraging iterative regression models to estimate plausible values under missing-at-random assumptions. Categorical variables with less than 10% missingness will undergo mode imputation, replacing gaps with the most frequent category, while variables exceeding this threshold will be excluded to avoid bias from unreliable estimations. This tiered approach balances data completeness with methodological rigor, preserving statistical validity while addressing real-world data imperfections.

#### 2.7.5 Data storage and security

To safeguard participant confidentiality, all patient identifiers will be removed and replaced with unique, non-traceable study IDs. Anonymized data will be stored in encrypted formats on password-protected servers hosted through the OSF platform (osf.io/ayj75), a secure repository compliant with international data protection standards. Access to the dataset will be restricted to authorized researchers via two-factor authentication, ensuring that only verified personnel with explicit permissions can retrieve or modify the data. This multi-layered security framework aligns with General Data Protection Regulation (GDPR) and China’s Data Security Law, prioritizing privacy while enabling rigorous scientific analysis. Lidström et al. also advocate the idea of securing patient data (37).

### 2.8 Statistical analysis

#### 2.8.1 Primary outcome analysis

The AUC will serve as the primary metric to evaluate the diagnostic accuracy of the AI model. To account for variability inherent in multi-reader, multi-case study designs, the DBM method will be employed, which adjusts for differences in clinician interpretations and case complexity. Analyses will be conducted using the MRMCaov (v0.2.1) and pROC (v1.18.0) packages in R, generating AUC values with 95% confidence intervals (CIs) derived from non-parametric bootstrapping (1,000 iterations). A clinically significant improvement is predefined as a ΔAUC more than 0.15 between the model-assisted and control groups, with the null hypothesis assuming no discriminative capacity (AUC less than 0.70, equivalent to random guessing). This approach ensures robust statistical inference while addressing real-world diagnostic variability across clinicians and settings. Hyperparameter optimization was conducted via Bayesian optimization with 5-fold cross-validation, prioritizing AUC on the validation set.

#### 2.8.2 Secondary outcomes analysis

Secondary endpoints include diagnostic sensitivity and specificity, calculated using the Clopper-Pearson exact method for binomial proportions to determine the model’s and clinicians’ ability to correctly identify true positive and true negative cases, respectively. Differences between clinician and model performance will be assessed via the Mann–Whitney U test, a non-parametric method suitable for skewed distributions. Positive and negative predictive values (PPV/NPV) will be adjusted for influenza prevalence using Bayes’ theorem and reported with 95% confidence intervals to reflect their clinical utility in real-world settings. The misdiagnosis rate, defined as the sum of false positives and false negatives per 100 diagnoses, will be analyzed using an independent samples t-test (if normally distributed) or Wilcoxon rank-sum test (for non-normal data), quantifying the model’s impact on reducing diagnostic errors. These metrics collectively evaluate the model’s precision, reliability, and practical value in augmenting clinical decision-making.

#### 2.8.3 Subgroup analyses

Subgroup analyses will evaluate the model’s performance heterogeneity across predefined categories: patient-level factors (age [infants, children, adults, older adults], comorbidities, and vaccination status), clinician-level factors (experience [resident, attending, chief] and hospital resource tier [urban vs. rural]), and geographic variation (northern vs. southern Jiangsu Province). To mitigate bias, statistical adjustments include Bonferroni correction for multiple comparisons (adjusted *α* = 0.05 / number of subgroups) and multivariable logistic regression controlling for confounders such as seasonal trends, baseline viral load, and treatment delays. These analyses aim to identify subgroups where the model excels or underperforms, ensuring tailored clinical deployment and validating robustness across diverse healthcare settings.

#### 2.8.4 Missing data handling

To address missing data, variables with more than 20% missing values (such as incomplete vaccination records) will be excluded to maintain analytical reliability. For remaining gaps, multiple imputation via chained equations (MICE package in R) will generate 10 imputed datasets for continuous variables, preserving statistical power under missing-at-random assumptions. Categorical variables with less than 10% missingness will undergo mode imputation, replacing missing entries with the most frequent category, while those exceeding this threshold will be excluded to avoid biased estimations. This protocol balances data completeness with methodological rigor, minimizing potential distortions in model performance evaluation.

#### 2.8.5 Sensitivity analyses

To evaluate the robustness of findings, sensitivity analyses will include complete case analysis (comparing results with and without imputation) and model stability assessments (quantifying AUC variation across imputed datasets). These analyses ensure conclusions are not unduly influenced by missing data assumptions. For computational workflows, R (v4.3.1), Python (v3.10), and Stata (v18) will be utilized, integrating specialized packages for imputation (MICE), AUC calculation (pROC), and regression modeling. Reporting will adhere to the Transparent Reporting of a multivariable prediction model for Individual Prognosis or Diagnosis (TRIPOD) guidelines for transparent validation of predictive models, ensuring methodological transparency, reproducibility, and alignment with international standards for AI-driven diagnostic research.

## 3 Ethics and dissemination

This study received ethical approval from the Ethics Committee of the Affiliated Hospital of Yangzhou University (Approval No. 2024-10-02). Annual renewals of ethical approval will be submitted to ensure ongoing compliance with evolving regulatory and ethical standards. Informed consent was obtained from all participants: patients or their legal guardians (for minors or incapacitated adults) provided written consent for anonymized data collection and analysis, while clinicians signed agreements acknowledging their roles in diagnosis and model interaction, with the explicit right to withdraw at any time. To safeguard privacy, all patient identifiers were replaced with unique study codes, and anonymized data were stored on the OSF platform (osf.io/ayj75), accessible only to authorized researchers via two-factor authentication. These measures align with international data protection standards and China’s Data Security Law, ensuring ethical integrity and participant confidentiality throughout the study.

### 3.1 Risk mitigation

To ensure patient safety and research integrity, clinicians retain full authority to override model recommendations if conflicting with clinical judgment, while an independent adjudication panel reviews all severe outcomes to confirm diagnostic accuracy. Emergency unblinding is permitted only when critical safety concerns necessitate full diagnostic transparency, with such instances documented and reviewed by ethics committees.

### 3.2 Dissemination plan

For broader dissemination, results will be shared across all 87 participating hospitals via workshops and a bilingual Clinical Decision Support Guide, with plans to integrate model outputs into EHR systems post-regulatory approval. Findings will be submitted to peer-reviewed journals and presented at international forums, complemented by public health bulletins highlighting implications for high-risk populations.

### 3.3 Data sharing policy

Data collected during the study will be stored under the supervision of the Ethics Committee of Yangzhou University Hospital. The China Clinical Research Registry advocates that data from clinical trials should be de-identified and then proactively disclosed, which is conducive to improving research transparency. However, we prefer to comply with the General Data Protection Regulation (GDPR) and China’s Data Security Law. This approach is conducive to gaining public trust and greatly protects the privacy of clinical trial participants. If a researcher is interested in the data, he or she should obtain ethical approval from the researcher’s institution and write a satisfactory and necessary research protocol. Once this has been done, the investigator can apply by email to the corresponding author. It is important to know that access involving sensitive human data must be subject to the jurisdiction and approval of the ethics committee. and Lidström et al. also advocate for the protection of patient data security (37).
